# *In silico* Analysis Reveals Distribution of Quorum Sensing Genes and Consistent Presence of LuxR Solos in the *Pandoraea* Species

**DOI:** 10.3389/fmicb.2019.01758

**Published:** 2019-08-06

**Authors:** Kah-Ooi Chua, Wah-Seng See-Too, Robson Ee, Yan-Lue Lim, Wai-Fong Yin, Kok-Gan Chan

**Affiliations:** ^1^Division of Genetics and Molecular Biology, Institute of Biological Sciences, Faculty of Science, University of Malaya, Kuala Lumpur, Malaysia; ^2^International Genome Centre, Jiangsu University, Zhenjiang, China

**Keywords:** cystic fibrosis, type strains, single molecule real-time sequencing, quorum sensing, LuxR solos

## Abstract

The most common quorum sensing (QS) system in Gram-negative bacteria consists of signaling molecules called *N-*acyl-homoserine lactones (AHLs), which are synthesized by an enzyme AHL synthase (LuxI) and detected by a transcriptional regulator (LuxR) that are usually located in close proximity. However, many recent studies have also evidenced the presence of LuxR solos that are LuxR-related proteins in Proteobacteria that are devoid of a cognate LuxI AHL synthase. *Pandoraea* species are opportunistic pathogens frequently isolated from sputum specimens of cystic fibrosis (CF) patients. We have previously shown that *P. pnomenusa* strains possess QS activity. In this study, we examined the presence of QS activity in all type strains of *Pandoraea* species and acquired their complete genome sequences for holistic bioinformatics analyses of QS-related genes. Only four out of nine type strains (*P. pnomenusa*, *P. sputorum*, *P. oxalativorans,* and *P. vervacti*) showed QS activity, and C8-HSL was the only AHL detected. A total of 10 canonical *luxI*s with adjacent *luxR*s were predicted by bioinformatics from the complete genomes of aforementioned species and publicly available *Pandoraea* genomes. No orphan *luxI* was identified in any of the genomes. However, genes for two LuxR solos (LuxR2 and LuxR3 solos) were identified in all *Pandoraea* genomes (except two draft genomes with one LuxR solo gene), and *P. thiooxydans* was the only species that harbored no QS-related activity and genes. Except the canonical LuxR genes, LuxIs and LuxR solos of *Pandoraea* species were distantly related to the other well-characterized QS genes based on phylogenetic clustering. LuxR2 and LuxR3 solos might represent two novel evolutionary branches of LuxR system as they were found exclusively only in the genus. As a few *luxR* solos were located in close proximity with prophage sequence regions in the genomes, we thus postulated that these *luxR* solos could be transmitted into genus *Pandoraea* by transduction process mediated by bacteriophage. The bioinformatics approach developed in this study forms the basis for further characterization of closely related species. Overall, our findings improve the current understanding of QS in *Pandoraea* species, which is a potential pharmacological target in battling *Pandoraea* infections in CF patients.

## Introduction

Cystic fibrosis (CF) results from mutations in the cystic fibrosis transmembrane conductance regulator (CFTR) gene that functions in modulating chloride ion transport across epithelial cells (Trapnell et al., 1991; Pier et al., 1996). As a consequence to this gene abnormality, majority of CF patients suffer from secretion of thick and viscous mucus in their respiratory tracts. These copious respiratory secretions become the breeding ground for microorganisms, which lead to both chronic and transient pulmonary infections, inflammation, obstruction of airways, and ultimately life-threatening pulmonary dysfunction (Gibson et al., 2003). *Staphylococcus aureus*, *Pseudomonas aeruginosa*, *Burkholderia cepacia,* and a spectrum of other Gram-negative bacteria are frequently associated with bacterial lung infections in CF patients (Gibson et al., 2003; LiPuma, 2010). However, recent reports revealed unprecedented infections by a number of bacteria and *Pandoraea* species are among the novel bacteria associated with pulmonary infections in CF patients (Atkinson et al., 2006; Davies and Rubin, 2007).

The genus *Pandoraea* was proposed to accommodate a group of isolates cultured from sputum specimens of CF patients that were initially misidentified as *B. cepacia* and genus *Ralstonia*. In the process of taxonomical characterization, some members of genus *Burkholderia* are reclassified into *Pandoraea* based on genotypic characteristics as well (Coenye et al., 2000, 2001). Members of genus *Pandoraea* are commonly recovered from sputum specimens of patients with cystic fibrosis, but some species were isolated from various environmental sources too. These bacteria have been considered as emerging multi-drug resistant pathogens in the context of cystic fibrosis (Davies and Rubin, 2007), but our understanding about the epidemics of *Pandoraea* species remains scarce.

Bacterial cells are able to interact with one another *via* production and release of diffusible signaling molecules into their living environment. Detection of such molecules enables bacteria to coordinate gene expression in response to both high and low cell population densities. The process is termed as quorum sensing (QS) or bacterial cell-to-cell communication (Williams, 2007). The canonical LuxI/R QS system is one of the most studied QS systems in bacteria. In this system, the responsible signaling molecules are *N*-acyl homoserine lactones (AHLs) that are produced by an AHL synthase, LuxI activates a cognate transcriptional regulator, LuxR if the concentration of AHLs achieves a threshold. Upon activation, LuxR binds to the promoters or regulators of targeted genes in response to the cell density and causes coordinated gene expression in the bacterial population (Fuqua and Greenberg, 2002; Williams, 2007). *Via* this system, bacteria regulate a variety of activities including biofilm formation, production of extracellular enzymes, regulation of virulence genes, and so on.

With the advancement in DNA sequencing technologies, novel subgroups of *luxI* and *luxR* homologs have been identified in numerous bacterial species. While it led to reports that most typical *luxI*/*R* QS systems have both genes involved located almost adjacent to each other, additional *luxR* homologs that do not pair with a cognate *luxI* are frequently found. These unpaired *luxR* homologs that are termed as *luxR* solos possess modular homologies to the canonical LuxR with an AHL-binding domain at their N-terminus and a DNA-binding helix-turn-helix (HTH) domain at the C-terminus (Subramoni and Venturi, 2009). Bioinformatics prediction of QS genes in proteobacterial genomes had revealed the presence of numerous additional orphan *luxR* homologs with no *luxI* homologs in close proximity (Fuqua, 2006). In addition to their widespread distributions in proteobacteria, some of these LuxR solos are phylogenetically related and several surveys provided evidence on clustering of LuxR solos into different functionally relevant groups (Brameyer et al., 2014; Gan et al., 2015; Subramoni et al., 2015). It is believed that the presence of additional LuxR solos increases the range of gene regulatory activities by responding to self-produced AHLs or eavesdropping on exogenous AHLs and even other signaling molecules produced by other species (Hudaiberdiev et al., 2015; Subramoni et al., 2015). Interestingly, some LuxR solos harbored by non-QS bacteria are responding to non-AHL signaling molecules such as OryR of *Xanthomonas oryzae* pv. oryzae and XccR of *Xanthomonas campestris* pv. campestris that are capable of interacting with plant signaling molecules and play essential roles in their pathogenicity (Zhang et al., 2007; Ferluga and Venturi, 2009).

Members of genus *Pandoraea* have been reported with QS activity and are able to communicate *via* the production of AHLs (Ee et al., 2014). In this study, we investigated (1) if AHL-mediated QS is a common activity employed by all type strains of *Pandoraea* species and (2) the distribution of QS genes in *Pandoraea* genus. Our work was initiated by obtaining type strains of all species of the genus from culture collection to characterize their QS activity before we sequenced their complete genomes to provide molecular data on distributions and phylogenetic relationships of QS genes in the study species. A systematic bioinformatics prediction workflow was developed for the identification of LuxI and LuxR of *Pandoraea* species. Our findings indicated that AHL synthases of genus *Pandoraea* represent a novel evolutionary branch of QS system. We also identified the presence of two conserved LuxR solos in most members of *Pandoraea* genus, which prompted us to further discuss the acquisition mechanisms and possible roles of these LuxR solos in this study.

## Materials and Methods

### Bacterial Strains and Culture Conditions

All type strains of species in *Pandoraea* genus were acquired from Leibniz Institute-Deutsche Sammlung von Mikroorganismen und Zellkulturen (DSMZ) culture collection. All bacteria strains were maintained in media and condition as listed in Supplementary Table S1.

### Detection of Quorum Sensing Activity Using CVO26 Bioassay

Preliminary detection of QS activity was conducted by CVO26 bioassay in which the bacterial samples were streaked perpendicularly to CVO26 biosensor on Luria-Bertani (LB) agar and incubated in 28°C for 24 h. The CVO26 biosensor is useful for the detection of short chain AHLs in the range of C4-HSL to C8-HSL (McClean et al., 1997). Positive result was observed with purple pigmentation of viocalein forming on the CVO26 biosensor. Positive and negative controls were set up with *E. carotovora* GS101 and *E. carotovora* PNP22, respectively.

### Extraction of Acyl-Homoserine Lactone Signaling Molecules

All the strains were cultured in LB broth buffered with 50 mM 3-(N-morpholino) propanesulfonic acid (MOPS; pH 5.5) in their respective optimum culturing temperature for 24 h with 220 rpm agitation prior to AHL extraction. Spent culture supernatants were mixed thoroughly with an equal volume of 0.1% v/v glacial acetic acid-acidified ethyl acetate solvent until biphasic layers were formed, and the upper immiscible solvent layer was transferred out. Similar extraction was performed twice, and the organic solvent containing AHL extract was desiccated completely for mass spectrometry analysis.

### Multiple Reaction Monitoring Mass Spectrometry Analysis

Desiccated AHL extracts were suspended with acetonitrile solvent prior to sample loading into an Agilent 1,290 Infinity LC system (Agilent Technologies Inc., Santa Clara, CA, USA). The liquid chromatography (LC) system was comprised of an Agilent ZORBAX Rapid Resolution High Definition SB-C18 Threaded Column (2.1 mm × 50 mm, 1.8 μm particle size) operated at 500 μl/min flow rate, 37°C with solvent A (0.1% formic acid buffered water) and solvent B (0.1% formic acid buffered acetonitrile) as the mobile phases. Three-step elution was performed with 7 min of linear gradient profile of 20–70% of solvent B, followed by 5 min of isocratic profile of 80% of solvent B, and 3 min of gradient profile of 80–20% of solvent B.

The LC-separated compounds were detected by electrospray ionization trap mass spectrometry (ESI-MS) using Agilent 6,490 Triple Quadrupole LC/MS system under positive-ion mode. The electrospray used nitrogen as nebulizing gas (pressure set to 20 p.s.i) and drying gas (flow set to 11 ml/h). The desolvation temperature was 200°C, and probe capillary voltage was set at 3 kV. AHL profiles were characterized using multiple reaction monitoring (MRM; Gould et al., 2006) by comparison of retention times and *m/z* transitions with those of the synthetic AHLs. A total of 10 synthetic AHLs varying in substitution oxo-group at C3 position (e.g., C6-HSL and 3-oxo-C6-HSL) and carbon length (ranging from C4-HSL to C12-HSL) were loaded in the MS analysis for reference as listed in Supplementary Table S2. The ions monitored in Q1 include the AHL precursor ion [M+H]^+^, whereas both the lactone moiety at *m*/*z* 102 and the acyl moiety [M+H−101]^+^ were monitored in Q3. Blanks (acetonitrile) were analyzed as control (data not shown). Data analysis was performed using Agilent MassHunter software.

### Genome Sequencing

Genomic DNA (gDNA) was extracted using MasterPure DNA Purification Kit (EpiCenter, CA, USA) according to the manufacturer’s instruction, and the quality of gDNA was assessed using gel electrophoresis, NanoDrop 2000 UV-Vis spectrophotometer (Thermo Scientific, MA, USA) and Qubit 2.0 fluorometer (Life Technologies, MA, USA), respectively. Whole genome sequencing was performed using PacBio (Pacific Biosciences, CA, USA) Single-Molecule Real Time (SMRT) sequencing technology.

### Genome Assembly, Circularization, and Annotation

Raw data generated were assembled using hierarchical genome assembly process (HGAP) assembler. Circularity of genomes was assessed using Contiguity (Sullivan et al., 2015; Lim et al., 2015a), and the precise location of the overlapping region was determined using Gepard (Krumsiek et al., 2007; Lim et al., 2015b) prior to genome circularization using Minimus2 pipeline in the AMOS software package to generate the blunt-ended circular genomes with complete closure. The presence of plasmid was distinguished from the chromosomal genome, and functional annotation was performed using NCBI Prokaryotic Genome Annotation Pipeline (PGAP), Rapid Prokaryotic Genome Annotation (Prokka; Seemann, 2014), Rapid Annotation Search Tool (RAST; Aziz et al., 2008), KEGG database (Kanehisa et al., 2016), and IMG ER (Markowitz et al., 2009).

### Systematic Bioinformatics Prediction of LuxI and LuxR

**Figure 1:**
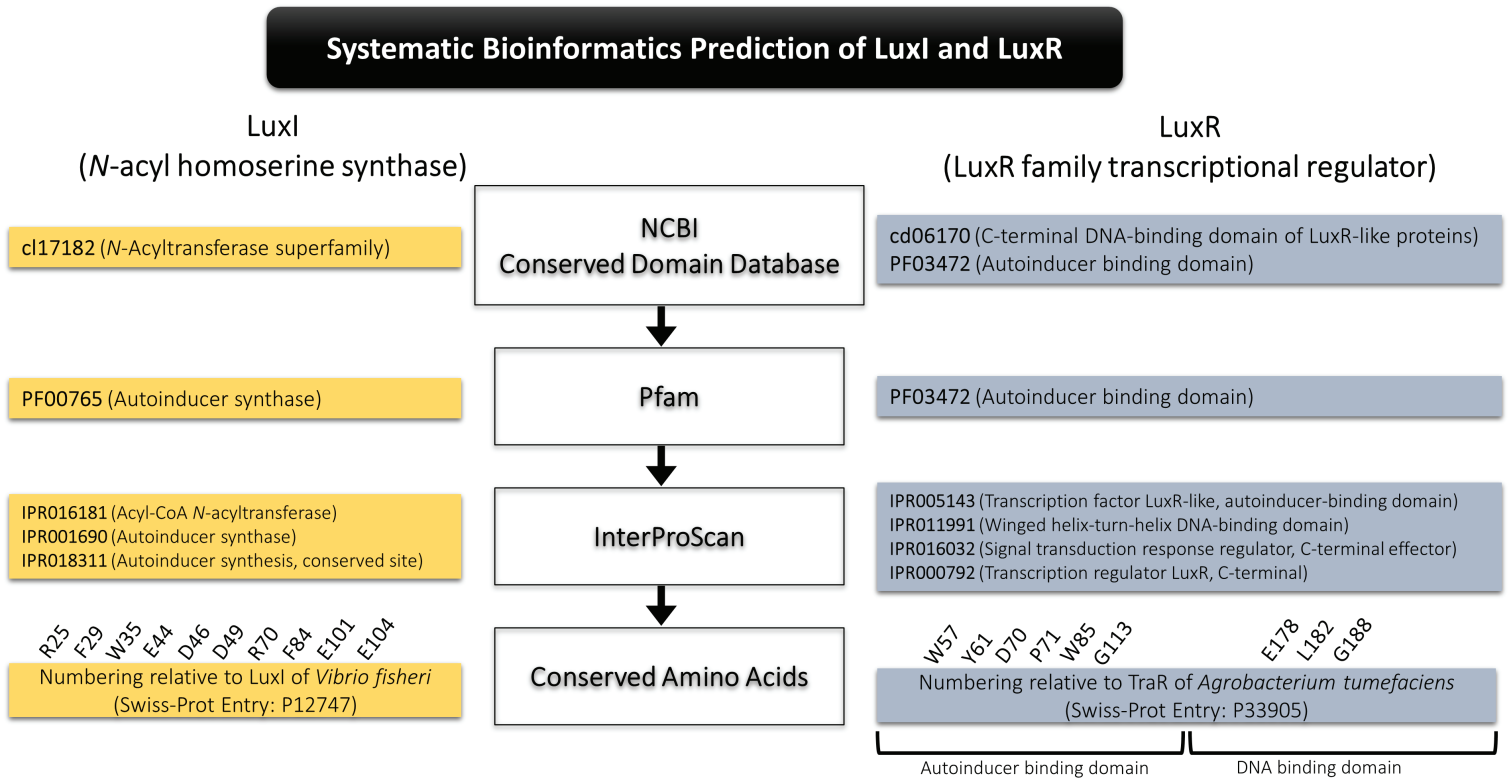
Systematic bioinformatics prediction of LuxI and LuxR in this study.

A systematic bioinformatics prediction and identification of LuxI and LuxR was employed in this study as presented in Figure 1. In short, translated proteomes were local blast against LuxI and LuxR databases downloaded from Uniprot and NCBI non-redundant protein database. Conserved domains of the putative LuxI (cl7182, N-acyltransferase superfamily) and LuxR (cd06170, C-terminal DNA-binding domain of LuxR-like proteins; PF03472, autoinducer-binding domain) were identified using conserved domain database (CDD) search tool in NCBI. All short-listed candidates were then scanned for signature protein family (Pfam) domain present in all LuxI (PF00765, autoinducer synthase), and LuxR (PF03472, autoinducer-binding domain). Lastly, a comprehensive InterProScan was conducted to provide high confidence authenticity of the identified LuxI and LuxR in which all LuxIs must contain three signature LuxI domains, IPR016181 (acyl-CoA N-acyltransferase), IPR001690 (autoinducer synthase), and IPR018311 (autoinducer synthesis, conserved site), while LuxR must contain four signature LuxR domains, IPR005143 (transcription factor LuxR-like, autoinducer-binding domain), IPR011991 (winged helix-turn-helix DNA-binding domain), IPR016032 (signal transduction response regulator, C-terminal effector), and IPR000792 (transcription regulator LuxR, C-terminal).

Multiple alignment of LuxI was performed with numbering relative to curated LuxI sequence of *Vibrio fischeri* (Entry: P12747) retrieved from Swiss-Prot database to determine the 10 conserved residues (R25, F29, W35, E44, D46, D49, R70, F84, E101, and E104) present in all LuxI (Fuqua and Greenberg, 2002). Multiple alignment of LuxR was performed with numbering relative to curated TraR sequence of *Agrobacterium tumefaciens* (Entry: P33905) retrieved from Swiss-Prot database to determine the nine signature conserved residues (W57, Y61, D70, P71 W85, and G113, which are key amino acids in autoinducer-binding domain, and E178, L182, and G188, which are three key amino acids in DNA-binding domain) found in all LuxR (Subramoni et al., 2015).

### Average Nucleotide Identity and Phylogenomics Analysis

Average nucleotide identity (ANI) analysis by Goris et al. (2007) was performed using ANI calculator^1^. Genome alignments were performed with a minimum length of 700 bp and a minimum identity of 70%. Genome fragments options were set with a window size of 1,000 bp and a step size of 200 bp. Phylogenomics tree was constructed using neighbor joining (NJ) method in MEGA 6.06 in which pairwise distances were acquired from ANI analysis (Saitou and Nei, 1987; Tamura et al., 2013).

### Maximum Likelihood Phylogenetic and Pairwise Identity Matrix Analyses

Evolutionary analyses of QS genes were performed using MEGA 6.06 in which all *in silico* functionally validated amino acid sequences were aligned using MUSCLE algorithm (Edgar, 2004; Tamura et al., 2013). Phylogenetic analyses were performed using maximum likelihood (ML) method with Jones-Taylor-Thorton (JTT) model and 1,000 replications of bootstrap analysis (Jones et al., 1992). Initial tree was constructed automatically using NJ and BioNJ algorithms with nearest-neighbor-interchange (NNI) method. All gaps and missing data were not included in the analyses. Pairwise identity matrix analyses were performed using Sequence Demarcation Tool (SDT) version 1.2 (Muhire et al., 2014). Sequences alignment was performed using MUSCLE algorithm, and data were presented in three color modes.

## Results and Discussion

### Detection and Characterization of Quorum Sensing Activity in *Pandoraea* Genus

Following our previous discovery on QS activity in *P. pnomenusa* RB-38 and RB-44 (Han-Jen et al., 2013; Ee et al., 2014), we hypothesized that QS could be a common activity employed by all members in *Pandoraea* genus. To prove our hypothesis, all nine type strains of *Pandoraea* were first screened for their QS activity using CVO26 biosensor (McClean et al., 1997), and other possible AHLs were also extracted from spent culture prior to characterization of AHL signaling molecules using MRM MS analysis. Of nine strains, only four (*P. pnomenusa, P. sputorum, P. oxalativorans,* and *P. vervacti*) activated the CVO26 biosensor, and C8-HSL was the only AHL detected in MRM MS analysis (Supplementary Figure S1). Interestingly, not all clinically isolated strains were detected positive for AHL production as *P. apista* DSM 16535^T^ and *P. pulmonicola* DSM 16583^T^ have no QS activity detected.

### Complete Genome Sequencing of Nine Type Strains of *Pandoraea* Species

As QS is not a common activity employed by all *Pandoraea* species, we questioned (1) if other non-AHL-producing *Pandoraea* species (*P. apista, P. pulmonicola, P. norimbergensis, P. faecigallinarum,* and *P. thiooxydans*) are actually possessing mutated *luxI* and/or *luxR*, incapable of producing or detecting AHL (Sandoz et al., 2007) and (2) if they harbor *luxR* solo in their genomes? To prove these hypotheses, we sequenced the complete genomes of all nine type strains of *Pandoraea* using SMRT sequencing technology to facilitate the identification of the QS genes in the genomes. HGAP assembler was employed to assemble all genomes to complete closure, and circularization was performed to provide a high confidence genomic size of each *Pandoraea* strains. Plasmids were distinguished from the chromosomal DNA, and designation code was provided for each strain (Table 1). Besides, all *Pandoraea* genomes available in GenBank were also retrieved for investigation (Supplementary Table S3).

**Table 1:** Designation code, sequencing information and general features of nine circularized genomes of *Pandoraea* type species.

Strains	Code	Assembler (coverage)	Chromosome				Plasmid			
			Accession no.	Genome size, bp	G + C content	Gene (protein)	Accession no.	Genome size, bp	G + C content	Protein (gene)
*P. pnomenusa* DSM 16536^T^	Ppn DSM	HGAP3 (244.6×)	CP009553.2	5,389,285	64.90	4,811 (4,586)	–	–	–	–
*P. pulmonicola* DSM 16583^T^	Ppu DSM	HGAP3 (110.5×)	CP010310.1	5,867,621	64.30	5,022 (4,820)	–	–	–	–
*P. sputorum* DSM 21091^T^	Psp DSM	HGAP3 (215.16×)	CP010431.1	5,751,958	62.80	5,037 (4,859)	–	–	–	–
*P. apista* DSM 16535^T^	Pap DSM	HGAP3 (243.8×)	CP013481.1	5,507,928	62.63	5,042 (4,864)	CP013482.1	77,293	62.63	5,042 (4,864)
*P. norimbergensis* DSM 11628^T^	Pno DSM	HGAP3 (165.0×)	CP013480.1	6,167,399	63.10	5,417 (5,195)	–	–	–	–
*P. faecigallinarum* DSM 23572^T^	Pfa DSM	HGAP3 (75×)	CP011807.1	5,261,138	63.70	4,619 (4,412)	CP011808.1 CP011809.1	402,292 124,395	61.00 59.30	388 (334) 130 (103)
*P. oxalativorans* DSM 23570^T^	Pox DSM	HGAP3 (215.2×)	CP011253.2	5,639,839	63.10	4,983 (4,711)	CP011518.1 CP011519.1 CP011520.1 CP011521.1	640,227 135,985 85,789 46,278	63.80 60.60 59.80 59.20	516 (452) 136 (120) 94 (70) 52 (40)
*P. thiooxydans* DSM 25325^T^	Pth DSM	HGAP3 (165.54×)	CP011568.1	4,464,186	63.20	4,117 (3984)	–	–	–	–
*P. vervacti* DSM 23571^T^	Pve DSM	HGAP2 (185×)	CP010897.1	5,656,222	63.50	4,912 (4697)	CP010898.1	105,231	62.00	102 (95)

Genome sizes of species in *Pandoraea* genus range between 4.5 and 6.2 Mb with genomes of *P. thiooxydans* DSM 25325 T and *P. norimbergensis* DSM 11628^T^ representing the smallest and largest, respectively (Table 1). The G + C content of these genomes varies from 62.63 to 64.9%. The presence of plasmids was identified in four of nine type strains. Notably, *P. apista* DSM 16535^T^ is the only one harboring plasmid of five clinically isolated strains. The other three type strains harboring plasmid are *P. faecigallinarum* DSM 23572^T^, *P. oxalativorans* DSM 23570^T^, and *P. vervacti* DSM 23571^T^ that were isolated from oxalate-enriched cultures from different environments (Table 1; Sahin et al., 2011).

**Figure 2:**
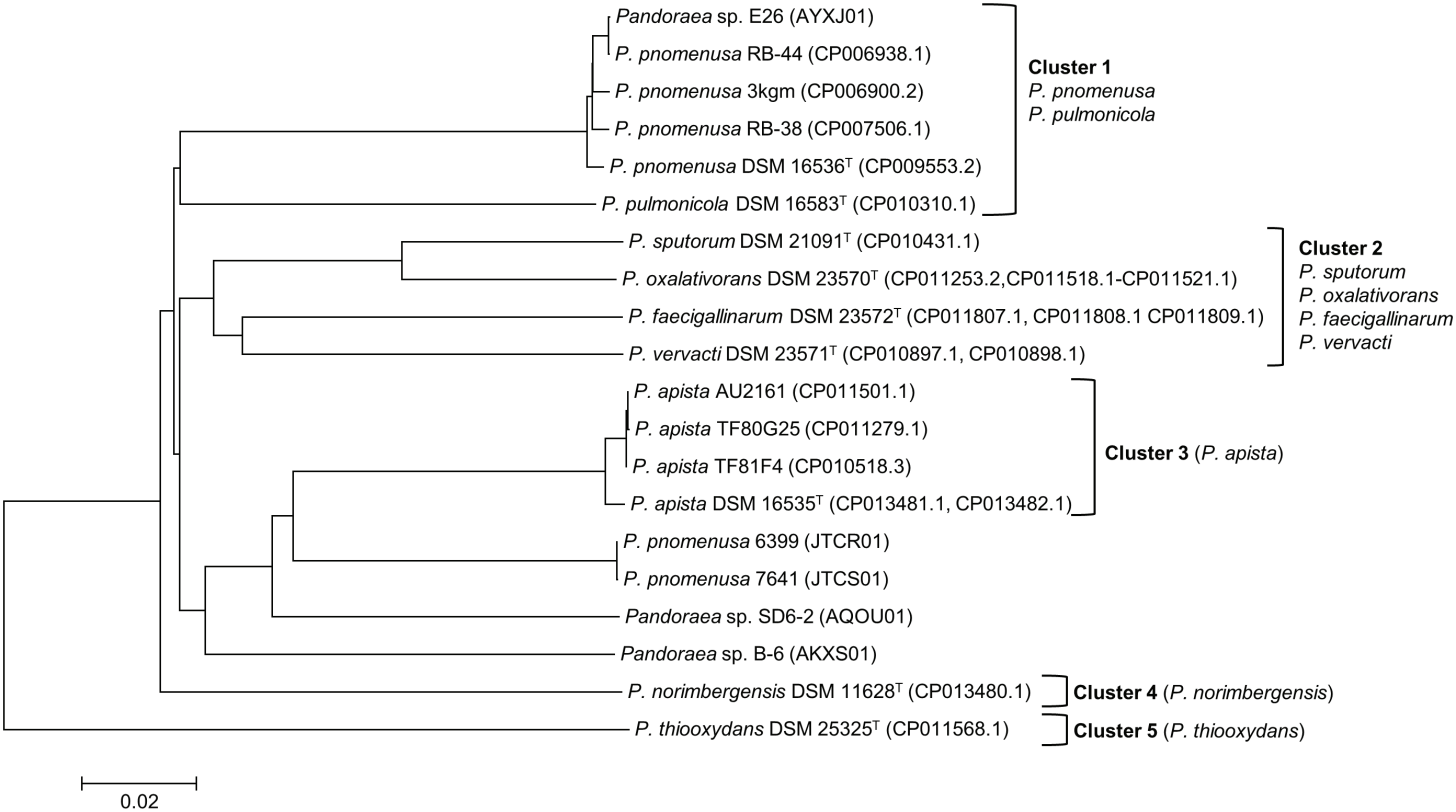
Phylogenomic analysis depicting the genetic and evolutionary distances of all *Pandoraea* species. In general, *Pandoraea* genus was separated into five distinct clusters: Cluster 1 (*P. pnomenusa*, *P. pulmonicola*), Cluster 2 (*P. sputorum*, *P. oxalativorans*, *P. faecigallinarum*, *and P. vervacti*), Cluster 3 (*P. apista*), Cluster 4 (*P. norimbergensis*), and Cluster 5 (*P. thiooxydans*). Bar, 0.2 substitutions per nucleotide position.

ANI analysis was subsequently performed to investigate the genetic and evolutionary distances of all *Pandoraea* species (Supplementary Table S4). Generally, 95% of ANI value is the accepted cut-off threshold for species-species delineation (Richter and Rosselló-Móra, 2009). The *Pandoraea* genomes in this study formed several clusters on a phylogenomic tree constructed using neighbor-joining algorithm (Figure 2). In Cluster 1 that included several *P. pnomenusa* and *P. pulmonicola* DSM 16583^T^, we observed that the clinically isolated strains *P. pnomenusa* DSM 16536^T^ were distinguished from the other *P. pnomenusa* that were obtained from the environments by slightly further distances.

By contrast, *P. sputorum* DSM 21091^T^ was the only clinically isolated species that was clustered together with *P. oxalativorans* DSM 23570^T^, *P. faecigallinarum* DSM 23572^T^, and *P. vervacti* DSM 23571^T^ that were isolated from various environments despite being obtained from different origins. The strains *P. norimbergensis* DSM 11628^T^ and *P. thiooxydans* were found to be most distantly related to all other *Pandoraea* species (<85 and <79% ANI values, respectively; Supplementary Table S4) forming outgroups in the phylogenomic analysis of *Pandoraea* genus (Figure 2).

Results from the ANI analysis also suggested reclassification of several *Pandoraea* strains with uncertain taxonomic status. We deduce from the analysis that *Pandoraea* sp. E26 could be reclassified as *P. pnomenusa* E26 as it shares 99% ANI value with all *P. pnomenusa*. On the other hand, *P. pnomenusa* strains 6,399 and 7,641 that demonstrated ANI value <90% against all other *P. pnomenusa* suggested that reclassification might be necessary. This is supported by the observations that both the strains exhibited the highest ANI values with *P. apista* species (>88% ANI values; Supplementary Table S4) and were placed out of Cluster 1 (consisted of all other *P. pnomenusa* and *P. pulmonicola* DSM 16583^T^) in phylogenomic tree (Figure 2). Unfortunately, the taxonomy of *Pandoraea* strains SD6-2 and B-6 remained questionable as both of them were having low ANI values (<88 and <86%, respectively; Supplementary Table S4) with all other *Pandoraea* in this study.

### Identification of *luxI* and *luxR* Homologs in Genomes of Genus *Pandoraea*

**Figure 3:**
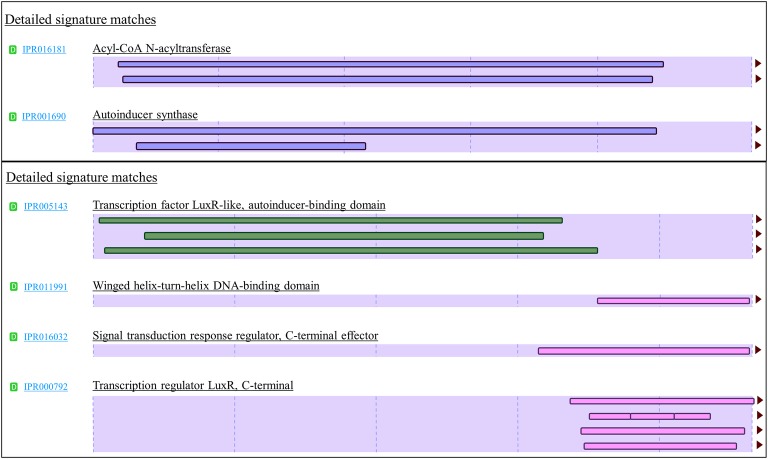
Signature InterPro domain of LuxI (top) and LuxR (bottom). LuxI of *Pandoraea* species contain two signature domains (IPR016181 and IPR001690), while LuxR of *Pandoraea* species contains four signature domains (IPR005143, IPR011991, IPR016032, and IPR000792).

To provide high confidence in authenticity of all LuxI and LuxR identified in this study, we created a stringent and effective systematic bioinformatics prediction of LuxI and LuxR as presented in Figure 1. A total of 10 *luxI*s were identified in genomes of *P. pnomenusa, P. sputorum, P. oxalativorans,* and *P. vervacti* (Supplementary Table S5). A typical authentic LuxI contains three signature InterPro domains (IPR016181, IPR001690, and IPR018311) and 10 signature conserved residues. Although all 10 LuxIs of *Pandoraea* species identified were found to contain only domains IPR016181 and IPR001690 (Figure 3), our previous gene cloning data of *PpnI* RB38 confirmed that LuxI of *Pandoraea* species could function properly despite the absence of domain IPR018311 (Lim et al., 2015a). Multiple alignment analysis of LuxI also revealed a consistent profile of signature conserved residues in all LuxIs of *Pandoraea* species, thus concordantly supported the evidence that these are authentic functional LuxI for the production of C8-HSL (Supplementary Table S6). No orphan *luxI* was identified in any of the *Pandoraea* genomes in this study.

Intriguingly, besides the expected canonical *luxR*, two additional *luxR* solos (named *luxR*2 solo and *luxR*3 solo) were identified in most *Pandoraea* genomes (Supplementary Table S5). *P. thiooxydans* DSM 25325^T^ is the only exception and does not harbor any canonical *luxI*/*R1* and *luxR* solo in its genome (Supplementary Table S5). *P. apista* TF81 and *Pandoraea* sp. E26 were also found to harbor only *luxR2* solo. However, we hypothesized that *luxR*3 solo could be missing in the gap of their draft genomes. All canonical LuxR and LuxR solos identified in this study contained all the four signature InterPro domains (IPR005143, IPR011991, IPR016032, and IPR000792), and all nine signature conserved residues (six key amino acids in autoinducer-binding domain and three key amino acids in DNA-binding domain) found in typical LuxR (Supplementary Table S7; Subramoni et al., 2015).

To date, there have been reports on LuxR solos responding to non-AHL signals. For examples, the PluR of *Photorhabdus luminescens* senses α-pyrone (Brachmann et al., 2013), while PauR of *P. asymbiotica* detects dialkylresorcinols and cyclohexanediones (Brameyer et al., 2015) signaling molecules instead of AHLs. These non-AHL-binding LuxRs, however, harbor substitutions in the conserved amino acid motif of autoinducer-binding domain compared to that in AHL sensors (Brameyer and Heermann, 2015). The autoinducer-binding domain of all LuxR solos in *Pandoraea* species contained the six conserved amino acids (W57, Y61, D70, P71, W85, and G113) with respect to TraR (Supplementary Table S7) and thus reflected a conserved motif for AHL-binding LuxR proteins.

From our analysis, we noticed that majority of the annotation pipelines often annotated *luxR*2 and *luxR*3 solo genes as hypothetical proteins making it a challenge in their identification process. Hence, we employed an *in silico* systematic bioinformatics prediction of these genes to aid in future identification of *luxR*2 and *luxR*3 solos. For nomenclature purpose, the gene products of canonical *luxI*/*R* identified in *Pandoraea* genomes were given designation with the first alphabet of the genus followed by the first two alphabet of species, for instances, PpnI, LuxI of *P. pnomenusa,* and PspR, LuxR of *P. sputorum* (Supplementary Table S5). Additionally, to differentiate between canonical LuxR and LuxR solos, canonical LuxR were given designation as LuxR1 (e.g., PpnI/R1 and PspI/R1), while LuxR solos were given designation as LuxR2 and LuxR3 solos. Gene designations, accession numbers, amino acid length, GC content, and genetic orientation of all canonical LuxI/R1 and LuxR solos of *Pandoraea* species are presented in Supplementary Table S5. No QS gene was found on plasmid.

### LuxI of *Pandoraea* Species Represents a Novel Evolutionary Branch of Quorum Sensing System

**Figure 4:**
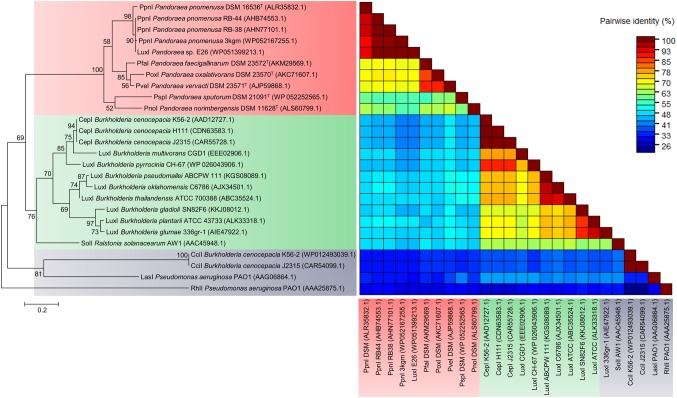
LuxI phylogenetic tree and pairwise identity matrix analyses of *Pandoraea* species and closely related species. LuxI of *Pandoraea* species form a distinct cluster against LuxI of *Burkholderia* species and *P. aeruginosa* representing an evolutionary distinct branch of QS system. Bootstrap values (expressed as percentages of 1,000 replications) greater than 50%. Bar, 0.2 substitutions per amino acid position.

To determine the relatedness of LuxI in *Pandoraea* genus with the other well-characterized LuxI, phylogenetic and pairwise identity matric analyses were conducted. Figure 4 shows the recent phylogenetic tree of LuxI from several groups that are most closely related to the LuxI in *Pandoraea* genus, together with pairwise identity matrix analysis. The analyses revealed that LuxI of *Pandoraea* species were highly conserved in *Pandoraea* genus forming a distinct cluster separated from the LuxI of *Burkholderia* species, SolI of *Ralstonia solanacearum*, and RhlI and LasI of *Pseudomonas aeruginosa*, representing a novel evolutionary branch of QS system (Figure 4). *Ralstonia* and *Burkholderia* are closely related genera to *Pandoraea*, and they shared highly similar phenotypic profiles that often resulted in the misidentification of *Pandoraea* species (Coenye et al., 2001; Henry et al., 2001). As *Pandoraea* were also predominantly recovered from CF patients, QS genes of *P. aeruginosa* (model organism for QS and CF patients) were also included in the analysis (Barr et al., 2015).

While analysis on the amino acid pairwise identity revealed that similarity of PpnI of different *P. pnomenusa* strains can be as high as 90%, pairwise identity shared between the LuxI of different *Pandoraea* species varied from about 50–90%, even though they were all placed in the same cluster in phylogenetic tree of LuxI (Figure 4). When compared to the LuxI of other genera, the LuxI of *Pandoraea* species are only 41–55% in pairwise identity with the well-characterized CepI of *Burkholderia cenocepacia* that catalyzes primarily the synthesis of C8-HSL and a minority of C6-HSL (Lewenza et al., 1999; Lewenza and Sokol, 2001); 48–55% in pairwise identity with SolI, which catalyzes the synthesis of C6-HSL and C10-HSL (Flavier et al., 1997); 26–41% in pairwise identity with CciI, which catalyzes primarily the synthesis of C6-HSL and a minority of C8-HSL (Malott et al., 2005); and lastly, about 26–33% pairwise identity with RhlI and LasI, which catalyzes primarily the synthesis of C4-HSL and 3-oxo-C12-HSL (Latifi et al., 1996). Besides, we also identified the 20 bp lux box located in the upstream region of the *luxI* of *Pandoraea* species (Supplementary Figure S2). The lux box is a 20 bp palindromic sequence located upstream in the promoter region of *luxI,* which is required for the binding of AHL-activated LuxR (Lewenza et al., 1999). All *luxI* of *Pandoraea* species shared consensus in 14 of 20 lux box sequence (Supplementary Figure S2).

### Canonical LuxR1 and LuxR Solos in *Pandoraea* Genus

**Figure 5:**
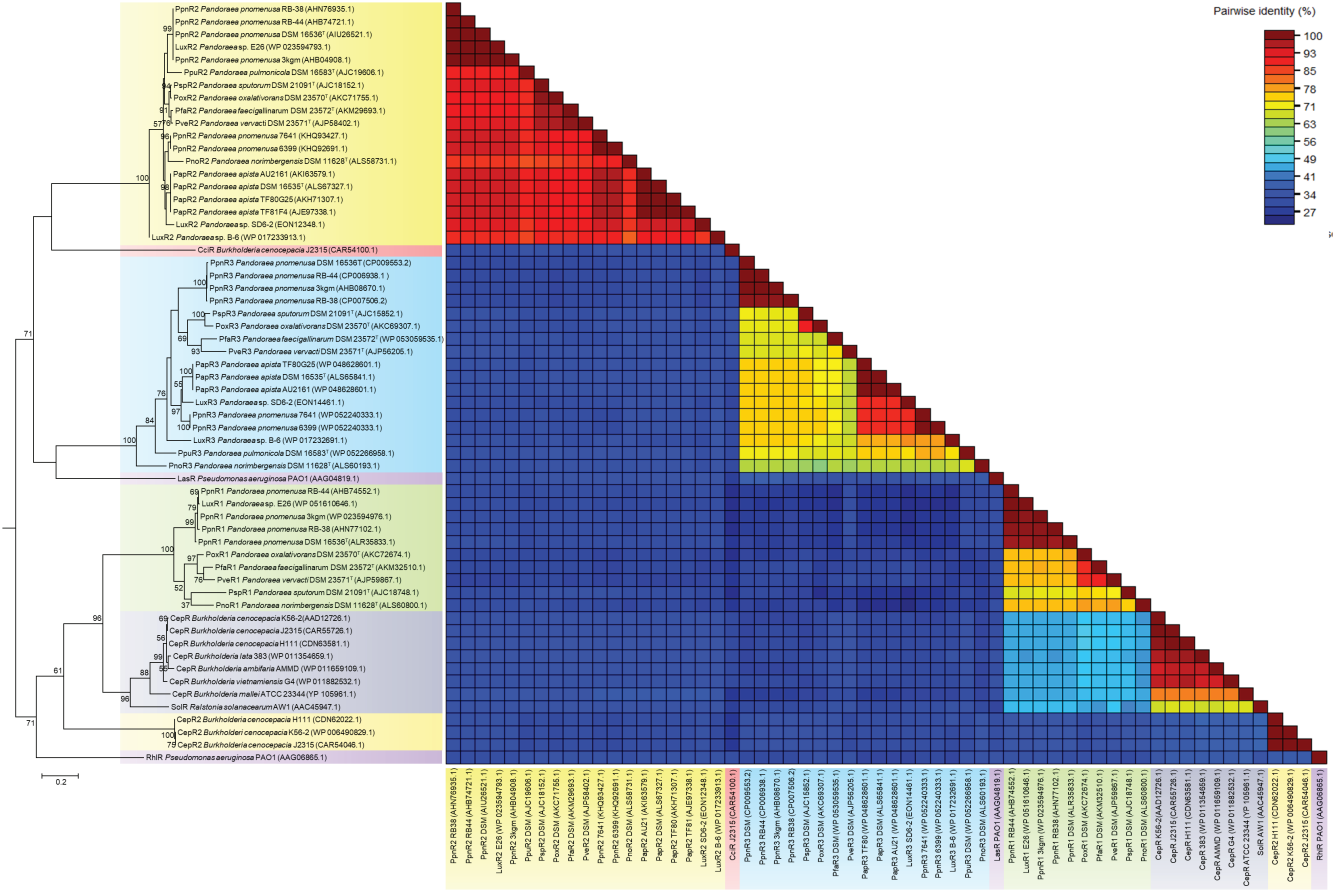
LuxR phylogenetic tree and pairwise identity matrix analyses of *Pandoraea* species and closely related species. Canonical LuxR1 clustered closely with CepR and SolR. LuxR2 and LuxR3 solos formed a distinct cluster with CciR and LasR as the outgroup, respectively, representing evolutionary distinct branches of LuxR. Bootstrap values (expressed as percentages of 1,000 replications) greater than 50%. Bar, 0.2 substitutions per amino acid position.

Phylogenetic and pairwise identity matric analyses performed on all identified canonical LuxR1 of genus *Pandoraea* demonstrated close clustering with CepR from genus *Burkholderia* and SolR from genus *Ralstonia* with 96% bootstrap value (Figure 5). Similar to CepI of genus *Burkholderia*, the LuxI of *Pandoraea* produce C8-HSL, but SolI from *Ralstonia* produces two short-chain AHL signals (C6-HSL and C10-HSL). As all identified *luxR*1 of *Pandoraea* are located adjacent to *luxI*, it is believed that the primary function of LuxR1 is for the detection of C8-HSL produced by its canonical LuxI. A comparison on the amino acid sequences of multiple LuxR groups revealed the highest pairwise identity among canonical LuxR1 of *Pandoraea* (71–100%) but lower identity to all the LuxR of other groups (<41%), including the LuxR2 and LuxR3 solos in *Pandoraea*.

Intriguingly, LuxR2 and LuxR3 solos formed two separated clusters on phylogenetic tree with the canonical LuxR identified in *Pandoraea* genus. Both the LuxR solos were distinctive from each other and had CciR of *Burkholderia cenocepacia* and LasR of *P. aeruginosa* as outgroups of the clusters, respectively (Figure 5). The LuxR2 solos are highly conserved in the genus *Pandoraea* showing >85% in amino acid pairwise identity among different species, as compared to the canonical LuxR1 (>71% in pairwise identity) and LuxR3 solos (>56% in pairwise identity) (Figure 5). From the phylogenetic analysis, it might imply that these LuxR solos in *Pandoraea* represent two novel evolutionary branches of LuxR in QS system. This is supported by a comprehensive search in various databases, which did not return significant matches with any other species and thus indicated that LuxR2 and LuxR3 solos were found exclusively only in *Pandoraea* species.

The widespread distribution of LuxR solos in almost every *Pandoraea* species (except *P. thiooxydans* DSM 25325^T^) indicated that they could be playing potential roles in survival and persistence of these species. For *Pandoraea* species that possess QS activity (*P. pnomenusa, P. sputorum, P. oxalativorans,* and *P. vervacti*), additional LuxR solos could function in detecting endogenous AHL signals produced by the AHL synthase to increase the regulatory targets of the complete canonical LuxI/R QS system. Notably, QS positive *P. pnomenusa* and *P. sputorum,* which are clinically isolated, might possess LuxR solos for their survival and persistence in respiratory tracts of CF patients as well as regulation of virulence factors. Similar phenomenon was observed in QscR solo of *Pseudomonas aeruginosa,* which is a LuxR solo that responds to endogenous 3-oxo-C12-HSL produced by LasI to control the timing of AHL production in the species for regulating expression of virulence factors. A study on *qscR* mutant demonstrated that it is hypervirulent in killing its host indicating that QscR solo is important for efficient regulation of QS-mediated virulence factors (Chugani et al., 2001).

In addition, the LuxR solos in *Pandoraea* species could be essential for detecting exogenous AHLs produced by neighboring species, especially for *Pandoraea* species that do not own a LuxI/R AHL system. In this study, AHL production was not observed in *P. apista* DSM 16535^T^ and *P. pulmonicola* DSM 21091^T^ that were isolated from sputa of CF patients. The presence of LuxR solos in these strains could be responsible for eavesdropping by detecting exogenous AHL molecules produced by *P. aeruginosa* that is chronically colonizing the respiratory tracts of CF patients. It is also noteworthy that many Gram-negative bacteria with QS activity such as *Burkholderia* and *Ralstonia* are common pathogens causing lung infections in CF patients. In fact, there are bacteria that possess LuxR solos even though they do not harbor any type of AHL synthase such as *Escherichia coli* and *Salmonella enterica* serovar Typhimurium. These bacteria carry a LuxR homolog, and SdiA was reported able to detect and respond to AHL signaling molecules produced by other bacterial species to activate their gene expression (Ahmer, 2004).

### Comparative Gene Mapping of All Quorum Sensing Genes and Putative Acquisition Mechanism of LuxR Solos in Type Strains of *Pandoraea*

**Figure 6:**
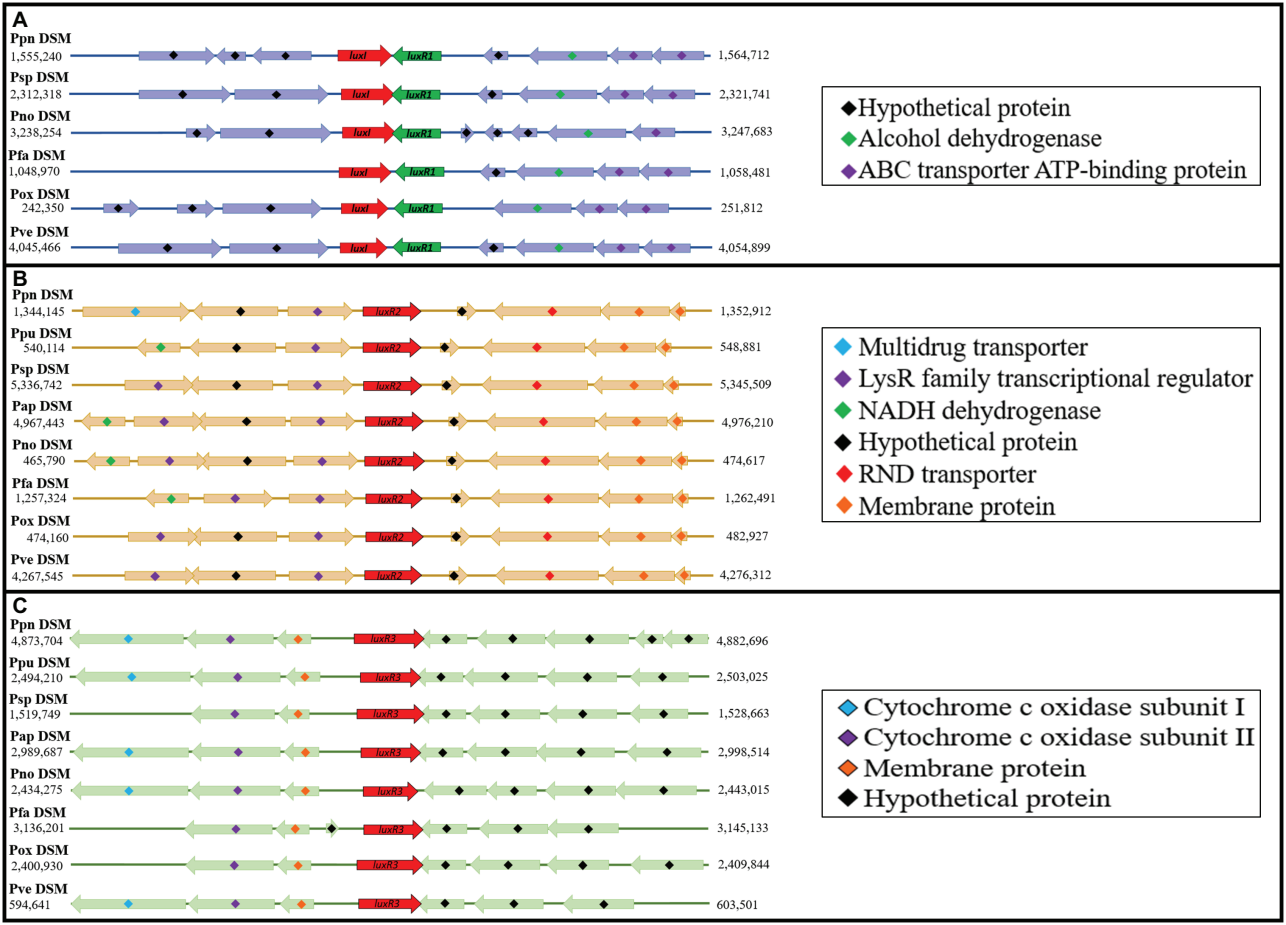
Comparative gene mapping of all QS genes in type strains of *Pandoraea* species. All QS genes were highly conserved at syntenic genomic location. **(A)** Canonical *luxI* and *luxR*1 were found be convergently inverted and located upstream of alcohol dehydrogenase and ABC transporter ATP-binding protein. **(B)** *luxR*2 solos located between LysR transcriptional regulators and RND transporter. **(C)** *luxR*3 solo located downstream of cytochrome c oxidase subunits I and II and a membrane protein.

Since this is the first documentation of *luxR*2 and *luxR*3 solos in *Pandoraea* genus, we are determined to investigate the acquisition mechanism of these genes in *Pandoraea* genus. Hence, we performed comparative gene mapping to study the degree of conservation of all QS genes. All QS genes of *Pandoraea* were found to be highly conserved at syntenic genomic locations (Figure 6): all canonical *luxI*/*R*1 were found to be convergently inverted (only *luxI*/*R*1 of *P. sputorum* and *P. norimbergensis* overlapped with each other) and located upstream of an alcohol dehydrogenase and an ABC transporter ATP-binding protein (Figure 6A); *luxR*2 solos were consistently located between a LysR transcriptional regulators and a RND transporter (Figure 6B); and *luxR*3 solos were always found located downstream of cytochrome c oxidase subunits I and II and a membrane protein (Figure 6C). As there are hypothetical proteins located in the immediate upstream of *luxR*2 and *luxR*3, we questioned if these hypothetical proteins could be the canonical *luxI* that have mutated and lost its function or domain. However, after a comprehensive domain prediction was performed on these hypothetical proteins, there was no residue of *luxI* in these hypothetical proteins.

Subsequently, we also performed an extensive search for the presence of any QS genes in genomic island, prophages, and mobile genetic element regions to determine the possibility of horizontal gene transfer event. No QS gene was found on any genomic island, and no residue of transposase was found in close proximity of all QS genes. Although no QS gene was found within any intact prophage region, there are, however, few *luxR* solos that were found in close proximity with incomplete and intact prophage sequences, such as 53,482 bp between PpuR2 (63.0% GC content; 544,114–544,881 bp) with an incomplete prophage region 1 (63.7% GC content; 597,363–605,870 bp); 62,330 bp between PoxR2 (60.0% GC content; 478,160–478,927 bp) with an incomplete prophage region 1 (62.91% GC content; 533,510–541,257 bp); and 6,231 bp between PoxR3 (65.2% GC content; 2,404,930–2,405,844 bp) with an intact prophage region 8 (63.2% GC content; 2,391,675–2,398,699 bp). These observations suggested that *luxR*2 and *luxR*3 solos could be transmitted into *Pandoraea* genus by transduction event mediated by prophage. However, parts of these prophage sequences might be lost during evolution. Various QS-related genes had been reported in the genomes of bacteriophages including homologs of accessory gene regulator (*agr*) in the genome of *Clostridium difficile* phage phiCDHM1 (Hargreaves et al., 2014) and regulatory protein LuxR in the *Azospirillum brasilense* Cd bacteriophage’s genome (Boyer et al., 2008).

QS activity in *Pandoraea* species has been related to the regulation of virulence factors, biofilm formation, extracellular enzymes production, antibiotic resistance, and various other lethal traits. Although not all *Pandoraea* species exhibit QS activity, findings in this study revealed that almost every *Pandoraea* species (except *P. thiooxydans* DSM 25325^T^) possess LuxR solos genes in their genomes. The repertoire of LuxR solos in the genus increases the range of gene regulatory activities and is anticipated to play roles in QS-dependent regulation of phenotypic functions, which should be investigated further. The data presented are also useful in future application including quorum quenching (QQ) study that attempts to disrupt the bacterial cell-to-cell communication of *Pandoraea* species through QS (See-Too et al., 2018). QQ has been suggested as alternative antibacterial strategy to antibiotics, which might lead to emergence of multi-drug resistant bacteria (Tang and Zhang, 2014). Last but not least, we hope that findings from this study contribute to further research to elucidate the downstream roles of QS genes in *Pandoraea* species, including their LuxR solos.

## Conclusions

Multiple species of the genus *Pandoraea* were frequently isolated from sputum samples of CF patients from all over the world, and *Pandoraea* species are identified as emerging pulmonary pathogen associated with CF. While some species were obtained from the environments, clinically isolated species such as *P. pnomenusa* has also been recovered from soils in the environment. This suggests the ubiquitous nature of this group of bacteria, and they are thus identified as opportunistic pathogens. The recent report on the QS activity in *P. pnomenusa* rapidly caught the attention of the scientific community as QS systems have been linked to the regulation of virulence factors, antibiotic resistance, and various traits that are dangerous to patients. Although this study revealed that only four type strains of nine species of genus *Pandoraea* possess AHL-based QS activity, we also reported the presence of two highly conserved *luxR* solos in most of their genomes. Our analyses had revealed that these LuxR solos belonged to different clusters of novel evolutionary branches in QS systems. We hypothesize that these LuxR solos in *Pandoraea* could potentially be responsive to AHLs or different signals produced by neighboring species and coordinate regulation of gene expression, thus playing important roles in the infection process and persistence of these pathogens in cystic fibrosis patients. In the process, we developed an *in silico* systematic bioinformatics prediction workflow, which is useful for LuxI and LuxR genes identification of other species. To summarize, this study lays the foundation for future study on QS systems of *Pandoraea* as a potential antimicrobial target in the treatment of *Pandoraea* infections.

## Data Availability

Publicly available datasets were analyzed in this study. This data can be found here: https://www.ncbi.nlm.nih.gov/genome/.

## Author Contributions

KG-C and WF-Y conceived and designed the experiment. RE and YL-L conducted the experiments. KO-C, WS-ST, RE, and YL-L conducted the data analyses. KO-C, WS-ST, and RE wrote the manuscript. All authors read and approved the manuscript.

### Conflict of Interest Statement

The authors declare that the research was conducted in the absence of any commercial or financial relationships that could be construed as a potential conflict of interest.

## Footnotes

^1^http://enve-omics.ce.gatech.edu/ani/
