# Supplementary material for: In silico Analysis Reveals Distribution of Quorum Sensing Genes and Consistent Presence of LuxR Solos in the Pandoraea Species
Source: Front Microbiol. 2019 Aug 6;10:1758. doi: 10.3389/fmicb.2019.01758 (PMC6691176; doi:10.3389/fmicb.2019.01758)
Supplement: Supplementary file 1 [file Data_Sheet_1.pdf]

Supplementary Materials

In silico analysis reveals novel class of quorum sensing genes and consistent presence of LuxR solos in the *Pandoraea* species

Kah-Ooi Chua[1], Wah-Seng See-Too[1], Robson Ee[1], Yan-Lue Lim[1], Wai-Fong Yin[1], Kok-Gan Chan[1,2*]

[1] Division of Genetics and Molecular Biology, Institute of Biological Sciences, Faculty of Science, University of Malaya, 50603 Kuala Lumpur, Malaysia.

[2] International Genome Centre, Jiangsu University, Zhenjiang, China

*** Correspondence:** Kok-Gan Chan: kokgan@um.edu.my

Supplementary Table S1. All bacteria strains with their description and culturing conditions used in this study.

Strain	Description	Culturing media, temperature	Source/reference
Chromobacterium violaceum CV026	Biosensor derived from double mini-Tn*5* mutant of ATCC31532 that synthesize purple violacein pigment for detection of short chain exogenous AHL.	LBA, 28°C	(McClean et al., 1997)
Erwinia carotovora GS101	Produces short chain exogenous AHL which served as positive control in CVO26 bioassay.	LBA, 28°C	Gift from Prof. Paul Williams
Erwinia carotovora PNP22	Negative control for CVO26 bioassay.	LBA, 28°C	Gift from Prof. Paul Williams
Pandoraea pnomenusa RB-38	Soil isolate	LBA, 28°C	This study
Pandoraea pnomenusa RB-44	Soil isolate	LBA, 28°C	This study
Pandoraea pnomenusa 3kgm	Soil isolate	LBA, 28°C	This study
Pandoraea pnomenusa DSM 16536^T	Recovered from cystic fibrosis patient, United Kingdom.	TSA, 37°C	(Coenye et al., 2000)
Pandoraea pnomenusa 6399	Recovered 11 months before the cystic fibrosis patient deceased, Australia.	TSA, 37°C	(Ee et al., 2015a)
Pandoraea pnomenusa 7641	Recovered from deceased cystic fibrosis patient, Australia.	TSA, 37°C	(Ee et al., 2015b)
Pandoraea pulmonicola DSM 16583^T	Recovered from sputum of cystic fibrosis patient, Canada.	TSA, 37°C	(Coenye et al., 2000)
Pandoraea sputorum DSM 21091^T	Recovered from sputum of cystic fibrosis patient USA.	TSA, 37°C	(Coenye et al., 2000)
Pandoraea apista DSM 16535^T	Recovered from sputum of cystic fibrosis patient, Denmark.	TSA, 37°C	(Coenye et al., 2000)
Pandoraea faecigallinarum DSM 23572^T	Isolated from chicken dung, India.	TSA, 28°C	(Sahin et al., 2011)
Pandoraea norimbergensis DSM 11628^T	Isolated from oxic water above sulfide containing lake sediment, Germany.	TSA, 37°C	(Coenye et al., 2000)
Pandoraea thiooxydans DSM 25325^T	Isolated from rhizosphere soils of sesame *Sesamum indicum* L., Korea.	TSA, 30°C	(Anandham et al., 2010)
Pandoraea vervacti DSM 23571^T	Isolated from uncultivated field soil, Turkey	TSA, 28°C	(Sahin et al., 2011)
Pandoraea oxalativorans DSM 23570^T	Isolated from soil litter close to *Oxalis* sp., Turkey.	TSA, 28°C	(Sahin et al., 2011)

Abbreviation:
LBA, Luria-bertani agar;
TSA, Tryptic soy agar

Supplementary Table 2. Precursor and product ion *m/z* values of synthetic AHLs.

**Synthetic AHL**	**Precursor ion, Q1 (*m/z*)**	**Product ion, Q3 (*m/z*)**
C4-HSL	172	102
C6-HSL	200	102
C7-HSL	214	102
C8-HSL	228	102
C10-HSL	256	102
C12-HSL	284	102
3-oxo-C6-HSL	214	102
3-oxo-C8-HSL	242	102
3-oxo-C10-HSL	270	102
3-oxo-C12-HSL	298	102

Supplementary Table 3. Designation code and accession number of all *Pandoraea* genomes retrieved from GenBank for this study.

Strain	Designation code	Finishing status	GenBank ID
P. pnomenusa RB-38	Ppn RB38	Complete genome	CP007506.3
P. pnomenusa RB-44	Ppn RB44	Complete genome	CP006938.1
P. pnomenusa 3kgm	Ppn 3kgm	Complete genome	CP006900.2
P. pnomenusa 6399	Ppn 6399	Draft genome	JTCR01
P. pnomenusa 7641	Ppn 7641	Draft genome	JTCS01
P. apista AU2161	Pap AU21	Complete genome	CP011501.1
P. apista TF80G25	Pap TF80	Complete genome	CP011279.1
P. apista TF81F4	Pap TF81	Complete genome	CP010518.3
Pandoraea sp. B-6	B-6	Draft genome	AKXS01
Pandoraea sp. E26	E26	Draft genome	AYXJ01
Pandoraea sp. SD6-2	SD6-2	Draft genome	AQOU01

Supplementary Table 4. ANI analysis between genomes of *Pandoraea* species.

		Ppn						Ppu	Psp	Pap				Pno	Pfa	Pox	Pth	Pve	*Pandoraea* sp.		
		DSM	RB38	RB44	3kgm	6399	7641	DSM	DSM	DSM	AU21	TF80	TF81	DSM	DSM	DSM	DSM	DSM	B-6	E26	SD6-2
Ppn	DSM	100	99.32	99.34	99.31	85.04	85.14	85.49	84.50	84.55	84.469	84.50	84.55	83.92	84.66	84.61	78.62	84.61	84.81	99.35	84.65
	RB38	99.32	100	99.41	99.44	84.66	84.59	85.06	84.50	84.43	84.48	84.46	84.40	83.97	84.52	84.71	78.57	84.67	84.77	99.41	84.67
	RB44	99.34	99.41	100	99.43	84.64	84.63	85.07	84.48	84.51	84.47	84.46	84.46	83.96	84.49	84.63	78.68	84.61	84.79	99.97	84.61
	3kgm	99.31	99.44	99.43	100	84.61	84.58	85.07	84.43	84.47	84.48	84.45	84.49	83.94	84.54	84.64	78.54	84.67	84.85	99.44	84.67
	6399	85.04	84.66	84.64	84.61	100	100	84.78	84.66	88.56	88.55	88.51	88.55	84.14	84.66	84.78	78.14	84.60	85.49	84.61	87.89
	7641	85.14	84.59	84.63	84.58	100	100	84.77	84.64	88.46	88.55	88.53	88.48	84.12	84.58	84.79	78.21	84.60	85.45	84.65	87.86
Ppu		85.49	85.06	85.07	85.07	84.78	84.77	100	84.68	84.60	84.55	84.58	84.60	84.33	84.63	84.80	78.79	84.73	85.05	85.10	84.75
Psp		84.50	84.50	84.48	84.43	84.66	84.64	84.68	100	84.55	84.54	84.64	84.55	83.80	85.85	92.39	78.37	85.56	84.60	84.41	84.71
Pap	DSM	84.55	84.43	84.51	84.47	88.56	88.46	84.60	84.55	100	99.31	99.31	99.31	84.15	84.58	84.68	78.19	84.44	85.32	84.44	87.77
	AU21	84.46	84.48	84.47	84.48	88.55	88.55	84.55	84.54	99.31	100	100	100	84.10	84.46	84.59	78.11	84.42	85.32	84.42	87.78
	TF80	84.50	84.46	84.46	84.45	88.51	88.53	84.58	84.64	99.31	100	100	100	84.10	84.48	84.72	78.11	84.48	84.30	84.48	87.77
	TF81	84.55	84.40	84.46	84.49	88.55	88.48	84.60	84.55	99.31	100	100	100	84.15	84.50	84.68	78.19	84.44	85.29	84.41	87.74
Pno		83.92	83.97	83.96	83.94	84.14	84.12	84.33	83.80	84.15	84.10	84.10	84.15	100	83.88	83.99	78.24	83.90	84.09	83.92	84.05
Pfa		84.66	84.52	84.49	84.54	84.66	84.58	84.63	85.80	84.58	84.46	84.48	84.50	83.80	100	85.10	78.47	86.70	84.54	84.47	84.62
Pox		84.61	84.71	84.63	84.64	84.78	84.79	84.80	92.39	84.68	84.59	84.72	84.68	83.99	85.72	100	78.35	85.90	84.71	84.60	84.79
Pth		78.62	78.57	78.68	78.54	78.14	78.21	78.79	78.37	78.19	78.11	78.11	78.19	78.24	78.47	78.35	100	78.25	78.08	78.42	77.85
Pve		84.61	84.67	84.61	84.67	84.60	84.60	84.73	85.56	84.44	84.42	84.48	84.44	83.90	86.70	85.90	78.25	100	84.53	84.49	84.59
sp.	B-6	84.81	84.77	84.79	84.85	85.49	85.45	85.05	84.60	85.32	85.32	84.30	85.29	84.09	84.54	84.71	78.08	84.53	100	85.05	85.81
	E26	99.35	99.41	99.97	99.44	84.61	84.65	85.10	84.41	84.44	84.42	84.48	84.41	83.92	84.47	84.60	78.42	84.49	85.05	100	84.67
	SD6-2	84.65	84.67	84.61	84.67	87.89	87.86	84.75	84.71	87.77	87.78	87.77	87.74	84.05	84.62	84.79	77.85	84.59	85.81	84.67	100

Supplementary Table 5. Gene designation, accession number, amino acid length, GC content and genetic orientation of all canonical LuxI/R1 and LuxR solos of *Pandoraea* species.

Strain	CVO26 bioassay (AHL detected)	Canonical LuxI Accession number (Length/GC content) Genetic orientation	Canonical LuxR1 Accession number (Length/GC content) Genetic orientation	LuxR2 solo Accession number (Length/GC content) Genetic orientation	LuxR3 solo Accession number (Length/GC content) Genetic orientation
Ppn DSM	Positive (C8-HSL)	PpnI<br>ALR35832.1<br>(245aa / 65.4%)<br>→	PpnR1<br>ALR35833.1<br>(233aa / 62.0%)<br>←	PpnR2<br>AIU26521.1<br>(255aa / 61.1%)<br>→	PpnR3<br>CP009553.2 (4877704bp - 4878696bp)<br>(330aa / 67.4%)<br>→
Ppn RB38	Positive (C8-HSL)	PpnI<br>AHN77101.1<br>(261aa / 65.5%)<br>→	PpnR1<br>AHN77102.1<br>(233aa / 62.0%)<br>←	PpnR2<br>AHN76935.1<br>(255aa / 61.0%)<br>→	PpnR3<br>CP007506.2 (4853186bp - 4854178bp)<br>(330aa / 67.3%)<br>→
Ppn RB44	Positive (C8-HSL)	PpnI<br>AHB74553.1<br>(261aa / 65.5%)<br>→	PpnR1<br>AHB74552.1<br>(233aa / 61.3%)<br>←	PpnR2<br>AHB74721.1<br>(255aa / 61.1%)<br>→	PpnR3<br>CP006938.1 (2823449bp – 2824450bp)<br>(333 aa / 67.5%)<br>→
Ppn 3kgm	ND	PpnI<br>WP_052167255.1<br>(191aa / 67.7%)<br>→	PpnR1<br>WP_023594976.1<br>(233aa / 61.5%)<br>←	PpnR2<br>AHB04908.1<br>(255aa / 61.1%)<br>→	PpnR3<br>AHB08670.1<br>(330aa / 67.3%)<br>→
Ppn 6399	ND	-	-	PpnR2<br>KHQ92691.1<br>(255aa / 59.1%)<br>→*	PpnR3<br>WP_052240333.1<br>(275aa / 65.0%)<br>→*
Ppn 7641	ND	-	-	PpnR2<br>KHQ93427.1<br>(255aa / 59.1%)<br>→*	PpnR3 (275 aa)<br>WP_052240333.1<br>(275aa / 65.0%)<br>→*
Ppu DSM	-	-	-	PpuR2<br>AJC19606.1<br>(255aa / 63.0%)<br>→	PpuR3<br>WP_052266958.1<br>(271aa / 68.4%)<br>→
Psp DSM	Positive (C8-HSL)	PspI<br>WP_052252565.1	PspR1<br>AJC18748.1	PspR2<br>AJC18152.1	PspR3<br>AJC15852.1

		(251aa / 64.4%)<br>→	(233aa / 57.3%)<br>←	(255aa / 60.4%)<br>→	(304aa / 64.6%)<br>→
Pap DSM	-	-	-	PapR2<br>ALS67327.1<br>(255aa / 57.9%)<br>→	PapR3<br>ALS65841.1<br>(275aa / 63.9%)<br>→
Pap AU21	ND	-	-	PapR2<br>AKI63579.1<br>(255aa / 57.9%)<br>→	PapR3<br>WP_048628601.1<br>(275aa / 63.9%)<br>→
Pap TF80	ND	-	-	PapR2<br>AKH71307.1<br>(255aa / 57.9%)<br>→	PapR3<br>WP_048628601.1<br>(275aa / 63.9%)<br>→
Pap TF81	ND	-	-	PapR2<br>AJE97338.1<br>(255aa / 57.9%)<br>→	Not found
Pno DSM	Negative<br>(ND)	PnoI<br>ALS60799.1<br>(249aa / 64.8%)<br>→	PnoR1<br>ALS60800.1<br>(237aa / 58.7%)<br>←	PnoR2<br>ALS58731.1<br>(275aa / 61.2%)<br>→	PnoR3<br>ALS60193.1<br>(246aa / 63.4%)<br>→
Pfa DSM	Negative<br>(ND)	PfaI<br>AKM29569.1<br>(248aa / 64.5%)<br>→	PfaR1<br>AKM32510.1<br>(233aa / 58.1%)<br>←	PfaR2<br>AKM29693.1<br>(255aa / 60.3%)<br>→	PfaR3<br>WP_053059535.1<br>(310aa / 68.5%)<br>→
Pox DSM	Positive<br>(C8-HSL)	PoxI<br>AKC71607.1<br>(222aa / 60.7%)<br>→	PoxR1<br>AKC72674.1<br>(233aa / 56.3%)<br>←	PoxR2<br>AKC71755.1<br>(255aa / 60.0%)<br>→	PoxR3<br>AKC69307.1<br>(304aa / 65.2%)<br>→
Pth DSM	-	-	-	-	-
Pve DSM	Positive<br>(C8-HSL)	PveI<br>AJP59868.1<br>(217aa / 62.8%)<br>→	PveR1<br>AJP59867.1<br>(233aa / 59.4%)<br>←	PveR2<br>AJP58402.1<br>(255aa / 58.9%)<br>→	PveR3<br>AJP56205.1<br>(286aa / 66.3%)<br>→
B-6	ND	-	-	LuxR2<br>WP_017233913.1<br>(255aa / 61.6%)<br>→*	LuxR3<br>WP_017232691.1<br>(275aa / 66.8%)<br>→*

E26	ND	LuxI<br>WP_051399213.1<br>(191aa / 68.1%)<br>→	LuxR1<br>WP_051610646.1<br>(233aa / 61.4%)<br>←	LuxR2<br>WP_023594793.1<br>(255aa / 61.1%)<br>→*	Not found
SD6-2	ND	-	-	LuxR2<br>EON12348.1<br>(255aa / 56.6%)<br>→*	LuxR3<br>EON14461.1<br>(275aa / 63.9%)<br>→*

Note:

Arterisk (*) indicates for unreliable orientation of *luxR2* solo and l*uxR3* solo that were retrieved from different contigs of a draft genome.

Supplementary Table 6. Multiple alignment analysis of LuxI of *Pandoraea* species. Numbering is relative to curated LuxI sequence of *Vibrio fisheri* (Entry: P12747) retrieved from Swiss-Prot database. Residues labelled with asterisk are residues whose mutation contributed significance loss in activity in LuxI and RhlI (Fuqua and Greenberg, 2002). PpnI 3kgm and LuxI E26 were retrieved from local PROKKA analysis as the sequences annotated by NCBI are truncated.

**LuxI**	**Key amino acid conserved in all LuxI-type protein**									
	**R25***	**F29**	**W35**	**E44***	**D46***	**D49***	**R70***	**F84**	**E101**	**R104***
PpnI DSM	R	F	W	E	D	D	R	F	E	R
PpnI RB38	R	F	W	E	D	D	R	F	E	R
PpnI RB44	R	F	W	E	D	D	R	F	E	R
PpnI 3kgm	R	F	W	E	D	D	R	F	E	R
PnoI DSM	R	F	W	E	D	D	R	F	E	R
PfaI DSM	R	F	W	E	D	D	R	F	E	R
PspI DSM	R	F	W	E	D	D	R	F	E	R
PoxI DSM	R	F	W	E	D	D	R	F	E	R
PveI DSM	R	F	W	E	D	D	R	F	E	R
LuxI E26	R	F	W	E	D	D	R	F	E	R

Supplementary Table 7. Multiple alignment analysis of LuxR of *Pandoraea* species. Numbering is relative to curated TraR sequence of *Agrobacterium tumefaciens* (Entry: P33905) retrieved from Swiss-Prot database (Subramoni et al., 2015).

**LuxR**		**Key amino acid in autoinducer binding domain**						**Key amino acid in DNA binding domain**		
		**W57**	**Y61**	**D70**	**P71**	**W85**	**G113**	**E178**	**L182**	**G188**
LuxR1	PpnR1 DSM	W	Y	D	P	W	G	E	L	G
	PpnR1 RB38	W	Y	D	P	W	G	E	L	G
	PpnR1 RB44	W	Y	D	P	W	G	E	L	G
	PpnR1 3kgm	W	Y	D	P	W	G	E	L	G
	PspR1 DSM	W	Y	D	P	W	G	E	L	G
	PnoR1 DSM	W	Y	D	P	W	G	E	L	G
	PfaR1 DSM	W	Y	D	P	W	G	E	L	G
	PoxR1 DSM	W	Y	D	P	W	G	E	L	G
	PveR1 DSM	W	Y	D	P	W	G	E	L	G
	LuxI E26	W	Y	D	P	W	G	E	L	G
LuxR2	PpnR2 DSM	W	Y	D	P	W	G	E	L	G
	PpnR2 RB38	W	Y	D	P	W	G	E	L	G
	PpnR2 RB44	W	Y	D	P	W	G	E	L	G
	PpnR2 3kgm	W	Y	D	P	W	G	E	L	G
	PpnR2 6399	W	Y	D	P	W	G	E	L	G
	PpnR2 7641	W	Y	D	P	W	G	E	L	G
	PpuR2 DSM	W	Y	D	P	W	G	E	L	G
	PspR2 DSM	W	Y	D	P	W	G	E	L	G
	PapR2 DSM	W	Y	D	P	W	G	E	L	G
	PapR2 AU21	W	Y	D	P	W	G	E	L	G
	PapR2 TF80	W	Y	D	P	W	G	E	L	G
	PapR2 TG81	W	Y	D	P	W	G	E	L	G
	PnoR2 DSM	W	Y	D	P	W	G	E	L	G
	PfaR2 DSM	W	Y	D	P	W	G	E	L	G
	PoxR2 DSM	W	Y	D	P	W	G	E	L	G
	PveR2 DSM	W	Y	D	P	W	G	E	L	G
	LuxR2 B-6	W	Y	D	P	W	G	E	L	G
	LuxR2 E26	W	Y	D	P	W	G	E	L	G
	LuxR2 SD6-2	W	Y	D	P	W	G	E	L	G
LuxR3	PpnR3 DSM	W	Y	D	P	W	G	E	L	G
	PpnR3 RB38	W	Y	D	P	W	G	E	L	G
	PpnR3 RB44	W	Y	D	P	W	G	E	L	G
	PpnR3 3kgm	W	Y	D	P	W	G	E	L	G
	PpnR3 6399	W	Y	D	P	W	G	E	L	G
	PpnR3 7641	W	Y	D	P	W	G	E	L	G
	PpuR3 DSM	W	Y	D	P	W	G	E	L	G
	PspR3 DSM	W	Y	D	P	W	G	E	L	G
	PapR3 DSM	W	Y	D	P	W	G	E	L	G
	PapR3 AU21	W	Y	D	P	W	G	E	L	G
	PapR3 TF80	W	Y	D	P	W	G	E	L	G
	PnoR3 DSM	W	Y	D	P	W	G	E	L	G
	PfaR3 DSM	W	Y	D	P	W	G	E	L	G
	PoxR3 DSM	W	Y	D	P	W	G	E	L	G
	PveR3 DSM	W	Y	D	P	W	G	E	L	G
	LuxR3 B-6	W	Y	D	P	W	G	E	L	G
	LuxR3 SD6-2	W	Y	D	P	W	G	E	L	G

Supplementary Figure 1. MRM MS analysis for characterization of AHL profile. Synthetic C8-HSL (*m/z*: 228) was loaded as the reference (top chromatogram). C8-HSL was detected as the only signaling molecule produced by *P. pnomenusa* DSM 16536^{T} (2nd chromatogram from top), *P. sputorum* DSM 21091^{T} (3rd chromatogram from top), *P. oxalativorans* DSM 23570^{T} (4th chromatogram from top) and *P. vervacti* DSM 23571^{T} (bottom chromatogram).

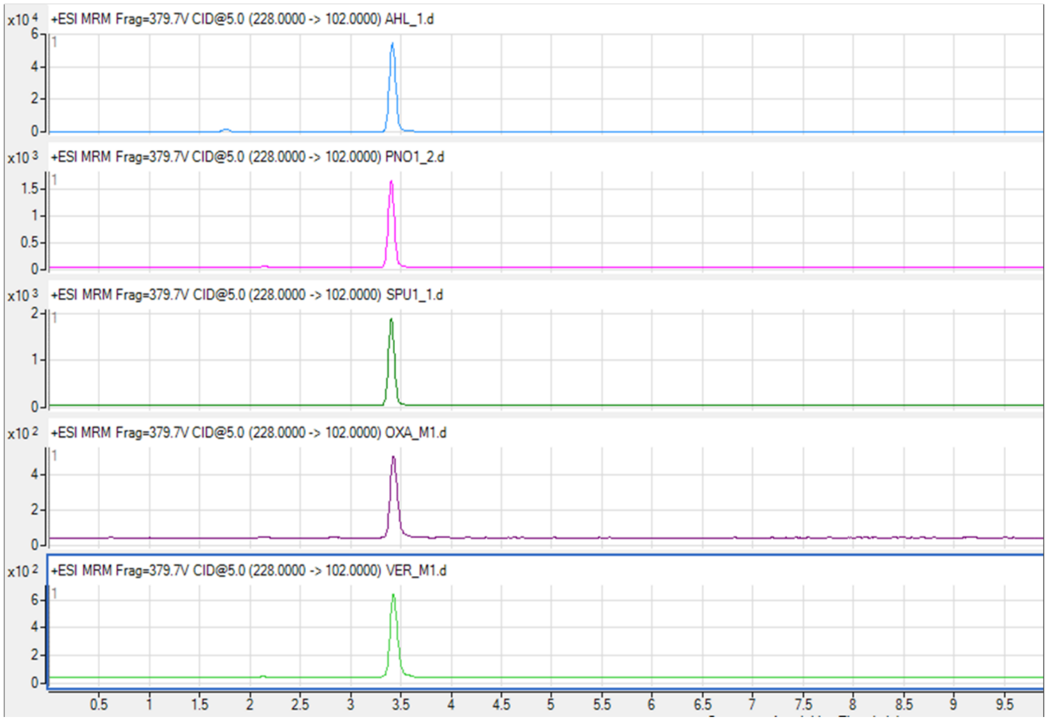

Supplementary Figure 2. Comparison of the 20bp *lux* box sequences located upstream of *cepI*, *solI*, *rhlI* and *luxI* of *Pandoraea* species. (**Figure 1a**) Sequences used for comparison are *cepI* K56-2, *Burkholderia cenocepacia* K56-2 (Lewenza et al., 1999); *solI* AW1, *Ralstonia solanacearum* AW1 (Flavier et al., 1997); *rhlI* PAO1, *Pseudomonas aeruginosa* PAO1 (Latifi et al., 1995). Black arrows indicate inverted repeats of the palindromic sequences and shaded boxes indicate identical nucleotide against the concensus sequence (Gray et al., 1994). Consensus sequence abbreviations are: N is A, T, C, or G; R is A or G; S is C or G; Y is T or C; and X is N or a gap in the sequence. (**Figure 1b**) Graphical representation of the *lux* box sequences.

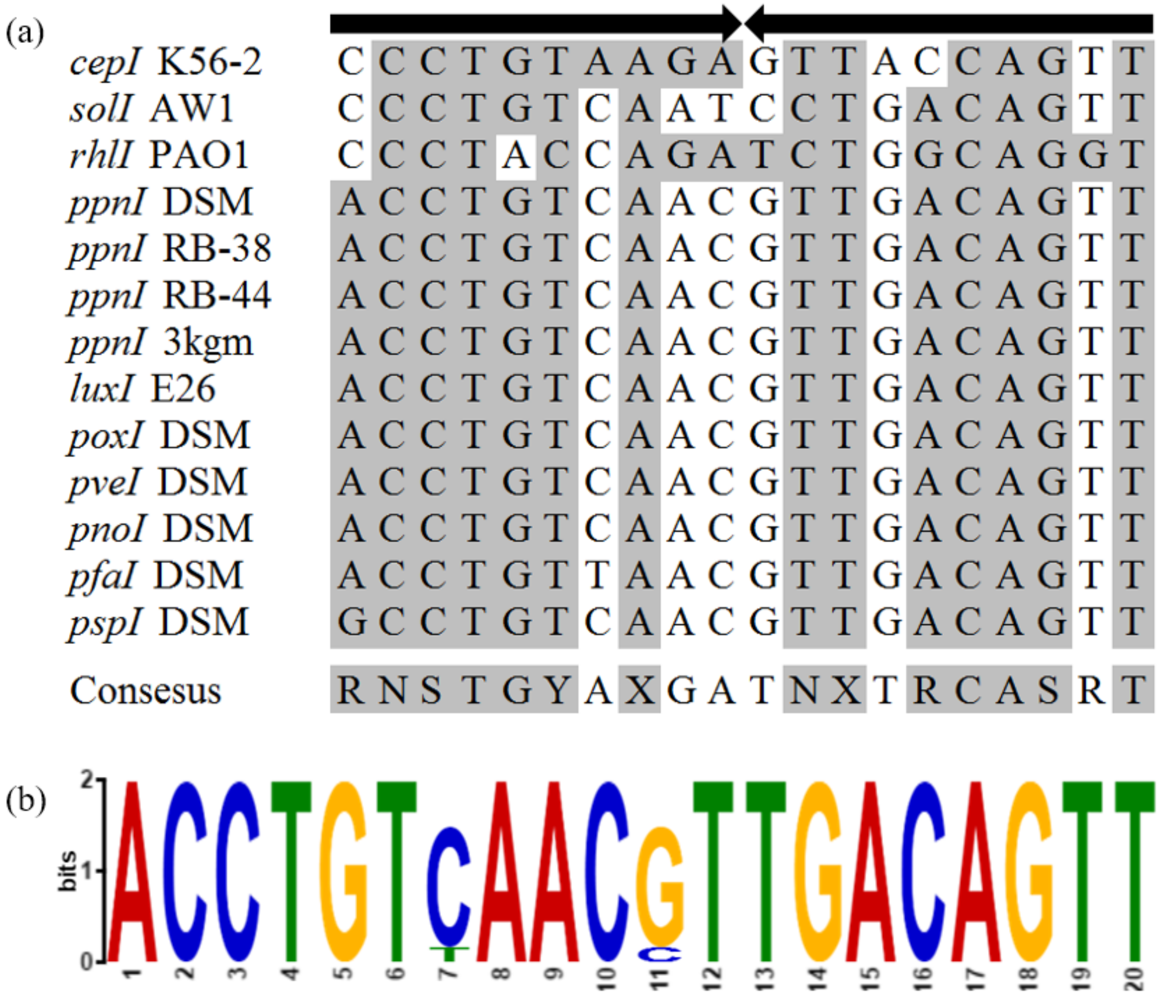

References

Anandham, R., Indiragandhi, P., Kwon, S.W., Sa, T.M., Jeon, C.O., Kim, Y.K., and Jee, H.J. (2010). *Pandoraea thiooxydans* sp. nov., a facultatively chemolithotrophic, thiosulfate-oxidizing bacterium isolated from rhizosphere soils of sesame (*Sesamum indicum* L.). *Int. J. Syst. Evol. Microbiol.* 60**,** 21-26.

Coenye, T., Falsen, E., Hoste, B., Ohlen, M., Goris, J., Govan, J.R., Gillis, M., and Vandamme, P. (2000). Description of *Pandoraea* gen. nov. with *Pandoraea apista* sp. nov., *Pandoraea pulmonicola* sp. nov., *Pandoraea pnomenusa* sp. nov., *Pandoraea sputorum* sp. nov. and *Pandoraea norimbergensis* comb. nov. *Int. J. Syst. Evol. Microbiol.* 50 Pt 2**,** 887-899.

Ee, R., Ambrose, M., Lazenby, J., Williams, P., and Chan, K.G. (2015a). Genome Sequences of Two *Pandoraea pnomenusa* Isolates Recovered 11 Months Apart from a Cystic Fibrosis Patient. *Genome Announc.* 3.

Ee, R., Ambrose, M., Lazenby, J., Williams, P., Chan, K.G., and Roddam, L. (2015b). Genome Sequences of Two *Pandoraea pnomenusa* Isolates Recovered 11 Months Apart from a Cystic Fibrosis Patient. *Genome Announc.* 3.

Flavier, A.B., Ganova-Raeva, L.M., Schell, M.A., and Denny, T.P. (1997). Hierarchical autoinduction in *Ralstonia solanacearum*: control of acyl-homoserine lactone production by a novel autoregulatory system responsive to 3-hydroxypalmitic acid methyl ester. *J. Bacteriol.* 179**,** 7089-7097.

Fuqua, C., and Greenberg, E.P. (2002). Listening in on bacteria: acyl-homoserine lactone signalling. *Nat. Rev. Mol. Cell. Biol.* 3**,** 685-695.

Gray, K.M., Passador, L., Iglewski, B.H., and Greenberg, E.P. (1994). Interchangeability and specificity of components from the quorum-sensing regulatory systems of *Vibrio fischeri* and *Pseudomonas aeruginosa*. *J. Bacteriol.* 176**,** 3076-3080.

Latifi, A., Winson, M.K., Foglino, M., Bycroft, B.W., Stewart, G.S., Lazdunski, A., and Williams, P. (1995). Multiple homologues of LuxR and LuxI control expression of virulence determinants and secondary metabolites through quorum sensing in *Pseudomonas aeruginosa* PAO1. *Mol. Microbiol.* 17**,** 333-343.

Lewenza, S., Conway, B., Greenberg, E.P., and Sokol, P.A. (1999). Quorum sensing in *Burkholderia cepacia*: identification of the LuxRI homologs CepRI. *J. Bacteriol.* 181**,** 748-756.

Mcclean, K.H., Winson, M.K., Fish, L., Taylor, A., Chhabra, S.R., Camara, M., Daykin, M., Lamb, J.H., Swift, S., Bycroft, B.W., Stewart, G.S., and Williams, P. (1997). Quorum sensing and *Chromobacterium violaceum*: exploitation of violacein production and inhibition for the detection of N-acylhomoserine lactones. *Microbiol.* 143 (Pt 12)**,** 3703-3711.

Sahin, N., Tani, A., Kotan, R., Sedlacek, I., Kimbara, K., and Tamer, A.U. (2011). *Pandoraea oxalativorans* sp. nov., *Pandoraea faecigallinarum* sp. nov. and *Pandoraea vervacti* sp. nov., isolated from oxalate-enriched culture. *Int. J. Syst. Evol. Microbiol.* 61**,** 2247-2253.

Subramoni, S., Florez Salcedo, D.V., and Suarez-Moreno, Z.R. (2015). A bioinformatic survey of distribution, conservation, and probable functions of LuxR solo regulators in bacteria. *Front. Cell. Infect. Microbiol.* 5**,** 16.
